# A cozy niche in an iron world

**DOI:** 10.1038/s41392-020-00368-4

**Published:** 2020-11-04

**Authors:** Marcus Conrad, Mariia Novikova

**Affiliations:** 1 Helmholtz Zentrum München, Institute of Metabolism and Cell Death, Ingolstädter Landstr. 1, 85764 Neuherberg, Germany; 2 Pirogov Russian National Research Medical University, Laboratory of Experimental Oncology, Ostrovityanova 1, Moscow, 117997 Russia; 3 Federal Center of Brain Research and Neurotechnology, Ostrovityanova1 bldg 10, 117997 Moscow, Russia

**Keywords:** Metastasis, Senescence

In this study, Ubellacker et al. show that unlike in blood vessels, the lymph is a more efficient route for tumor metastasis due to a more favorable redox environment, whereby disseminating cancer cells acquire additional protection against ferroptosis via incorporation of monosaturated fatty acids into their lipid bilayers.^1^

**Fig. 1:**
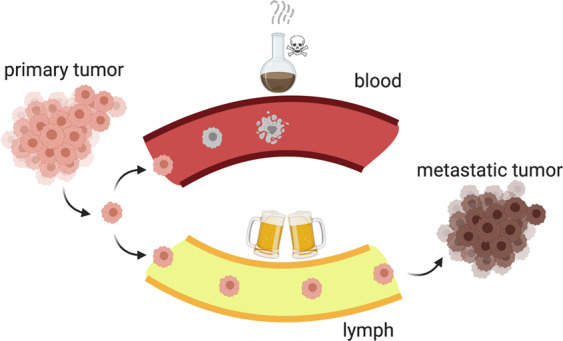
Due to a more favorable redox environment, tumor metastasis via the lymph is much more efficient than via the bloodstream

*Gemütlichkeit*—A German word without actual translation in other languages is used to describe a state of being “relaxed and cozy”. It usually refers to a unique atmosphere, for instance, when enjoying a couple of drinks with your mates in a comfy location like a beergarden. Seemingly, disseminating cancer cells when going on their long and lonesome journey to generate distant metastases prefer to travel via the lymphatics, a somewhat fairly friendly and “*gemütlich*” environment, rather than the bloodstream (Fig. 1). To avoid the harsh and potentially pro-ferroptotic conditions disseminating cancer cells may encounter, migrating cells not only choose the lymph as the prime route, but they also alter their membrane makeup as a cell-intrinsic mechanism to protect themselves against ferroptosis.

Ferroptosis is a metabolic form of cell death marked by an iron-dependent peroxidation of membrane polyunsaturated fatty acyl phospholipids. Accumulation of phospholipid hydroperoxides is generally prevented by the activity of the main ferroptosis suppressor, glutathione peroxidase 4 (GPX4), that catalyzes the glutathione (GSH)-dependent reduction of lipid hydroperoxides to their corresponding alcohols. While this enzyme is highly relevant for optimal functioning of normal cells, a growing body of evidence suggests a strong dependence on GPX4 for growth and survival of cancer cells.^2^ For instance, it was repeatedly shown that therapy-resistant cancer cells and those in a high-mesenchymal state become dependent on GPX4 activity and other enzymes involved in polyunsaturated fatty acid metabolism.^3^ This acquired dependence on a pathway protecting against lethal lipid peroxidation renders the therapy-resistant high-mesenchymal state particularly sensitive to ferroptosis, thus opening a new window for therapeutic intervention.

One may assume that increased ferroptosis could potentially account for the low survival rates of cancer cells metastasizing via the blood. Evidence supporting the involvement of ferroptosis in tumor metastasis has been provided in this study by Ubellacker et al.,^1^ whereby the authors have focused on comparing dissemination of metastasizing melanoma cells through blood versus lymphatic fluid. To this end, the authors injected mice with patient-derived or mouse melanoma cells and found that melanoma cells are by far more efficient in metastasis formation after intranodal injection than intravenous injection. Further experiments, in which melanoma cells were injected subcutaneously, revealed that cancer cells were more abundant in tumor-draining lymph nodes than in tumor-draining blood, suggesting a higher fitness and better survival of cancer cells in lymph than in blood. This apparent difference in the survival of circulating metastasizing cells was associated with an increased level of reactive oxygen species and a lower ratio of reduced versus oxidized GSH in cells disseminating in blood, indicating increased intracellular oxidative stress. This may result from a more oxidative and hostile environment in the blood, characterized by a higher concentration of free iron and a lower concentration of reduced GSH. Interestingly, cells that spontaneously metastasized through the blood also exhibited increased lipid peroxide formation. To ascertain that ferroptosis indeed contributes to impaired metastasis, melanoma cells were treated with a ferroptosis inhibitor, liproxstatin-1, a potent radical trapping antioxidant. While the extent of metastatic disease burden was not affected after intranodal transplantation of liproxstatin-1-pretreated cells, a substantial increase of metastasis was observed after intravenous injection, suggesting that ferroptosis restrains tumor metastasis via the blood. This was corroborated by experiments using mouse melanoma cells genetically engineered to lack *Gpx4*. The targeted knockout of *Gpx4* decreased the percentage of mice with formed metastatic tumors after intravenous injection of *Gpx4*-deleted melanoma cells, but not after intranodal injection. These results clearly demonstrate that cancer cells are prone to undergo ferroptosis and become dependent on GPX4 activity during metastasis in the blood.

To unravel additional cell-intrinsic mechanisms that might contribute to protection of cancer cells against ferroptosis in the lymph, the authors performed metabolomic and lipidomic analyses of metastasizing melanoma cells, and detected an enrichment of oleic acid in lymph-derived cells, a monounsaturated fatty acid previously described to confer robust antiferroptotic activity.^4^ This correlates with the finding that the levels of oleic acid-containing lipids were higher in lymph, where oleic acid is overwhelmingly present in triacylglycerols within ApoB-positive vesicles. Consequently, pretreatment of melanoma cells with oleic acid increased tumor metastasis after intravenous injection.^1^ Incorporation of oleic acid into cellular membranes was further shown to require the activity of a distinct fatty acid ligase, i.e., acyl-CoA synthetase long-chain family member 3. As a final proof-of-concept, the authors addressed whether a lymph environment provides any benefits for cancer cells to form subsequent distal metastases through the blood. To this end, cancer cells derived from lymph-node metastases were intravenously injected and they emerged to be more efficient in metastasis than intravenously injected control cells from the subcutaneous tumor. Besides, those cells were generally more resistant to a ferroptosis inducer erastin in vitro. Interestingly, the transient subcutaneous growth of the lymph-node-derived cells completely abrogated their ability to efficiently metastasize through blood, suggesting a reversible mechanism of the lymph-associated antiferroptotic phenotype.

Conclusively, the findings of this exciting study provide compelling evidence supporting the concept that lymph provides a more “cozy” and favorable environment for disseminating cancer cells, and after being exposed to that environment, the cells become more efficient in subsequent metastasis in the blood. This study may have far-fetched implications as the cystine/glutamate antiporter, known as system x_c_^−^, a heterodimeric amino acid transporter supplying cells with cysteine, the building block of GSH, is considered the most upstream component of ferroptosis. Besides its role in cysteine delivery to cells, system x_c_^−^ was also shown to be the driver of the cystine/cysteine redox cycle that is critically involved in generating a highly reducing environment that might also be favorable to nearby cells. In this context, a recent study showed that the targeted knockout of the substate-specific subunit of system x_c_^−^, xCT, in B16F10 melanoma cells almost completely abrogated tumor metastasis and consequently allowed drastically increased survival rates of tumor-bearing mice.^5^ The findings presented by this and earlier studies thus reinforce the notion that pharmacological targeting of xCT is perhaps one of the most promising strategies to combat disseminating tumor cells.
